# Reductions in microfilaridermia by repeated ivermectin treatment are associated with lower *Plasmodium*-specific Th17 immune responses in *Onchocerca volvulus*-infected individuals

**DOI:** 10.1186/s13071-015-0786-5

**Published:** 2015-03-28

**Authors:** Kathrin Arndts, Ute Klarmann-Schulz, Linda Batsa, Alexander Y Debrah, Christian Epp, Rolf Fimmers, Sabine Specht, Laura E Layland, Achim Hoerauf

**Affiliations:** Institute of Medical Microbiology, Immunology and Parasitology (IMMIP), University Hospital Bonn, Sigmund Freud Straße 25, Bonn, 53105 Germany; Institute of Medical Biometry, Informatics and Epidemiology (IMBIE), University Hospital Bonn, Bonn, Germany; Kumasi Centre for Collaborative Research in Tropical Medicine (KCCR), Kumasi, Ghana; Faculty of Allied Health Sciences of the Kwame Nkrumah University of Science and Technology, Kumasi, Ghana; Centre for Infectious Diseases - Parasitology, University Hospital Heidelberg, Heidelberg, Germany

**Keywords:** *Onchocerca volvulus*, Microfilariae, *Plasmodium*, Cytokine, Ivermectin, MSP-1, Multivariable regression analysis

## Abstract

**Background:**

37 million individuals are currently infected with *Onchocerca volvulus* (*O. volvulus*), a parasitic nematode that elicits various dermal manifestations and eye damage in man. Disease control is primarily based on distributing ivermectin in mass drug administration (MDA) programmes which aim at breaking transmission by eliminating microfilariae (MF), the worm's offspring. The majority of infected individuals present generalized onchocerciasis, which is characterized by hyporesponsive immune responses and high parasite burden including MF. Recently, in areas that have been part of MDA programmes, individuals have been identified that present nodules but are amicrofilaridermic (a-MF) and our previous study showed that this group has a distinct immune profile. Expanding on those findings we determined the immune responses of *O. volvulus*-infected individuals to a *Plasmodium-*derived antigen MSP-1 (merozoite surface protein-1), which is required by the parasite to enter erythrocytes.

**Methods:**

Isolated PBMCs from *O. volvulus*-infected individuals (164 MF^+^ and 46 a-MF) and non-infected volunteers from the same region (NEN), were stimulated with MSP-1 and the resulting supernatant screened for the presence of IL-5, IL-13, IFN-γ, TNF-α, IL-6, IL-17A and IL-10. These findings were then further analyzed following regression analysis using the covariates MF, ivermectin (IVM) and region. The latter referred to the Central or Ashanti regions of Ghana, which, at the time sampling, had received 8 or 1 round of MDA respectively.

**Results:**

IL-5, IL-13 and IFN-γ responses to MSP-1 were not altered between NEN and *O. volvulus*-infected individuals nor were any associations revealed in the regression analysis. IL-10, IL-6 and TNF-α MSP-1 responses were, however, significantly elevated in cultures from infected individuals. Interestingly, when compared to a-MF individuals, MSP-induced IL-17A responses were significantly higher in MF^+^ patients. Following multivariable regression analysis these IL-10, IL-6, TNF-α and IL-17A responses were all dominantly associated with the regional covariate.

**Conclusions:**

Consequently, areas with a lowered infection pressure due to IVM MDA appear to influence bystander responses to *Plasmodium*-derived antigens in community members even if they have not regularly participated in the therapy.

**Electronic supplementary material:**

The online version of this article (doi:10.1186/s13071-015-0786-5) contains supplementary material, which is available to authorized users.

## Background

Chronic filarial nematode infections in man are elicited through *Wuchereria bancrofti*, *Brugia* species, *Onchocerca volvulus*, *Loa loa* and *Mansonella* species which reside in subcutaneous tissues and lymphatics [1,2]. *O. volvulus* infections are primarily restricted to Africa and are recognized as the agents responsible for river blindness and various dermatological manifestations [3]. Infections are transmitted through the bite of *Simulium* black flies that release L3 stage larvae into the host during a blood meal. To complete the life-cycle, adult worms produce microfilariae (MF) which reside in the skin awaiting uptake by a further blood-feeding vector [1]. The principal goal of current mass drug administration (MDA) programmes is to break transmission by providing a yearly administration of ivermectin-based drug regimes [4-6]. IVM treatment, however, has only limited macrofilaricidal effects: successful elimination of adult worms can only be achieved through the administration of tetracycline antibiotics that target the essential endosymbiont *Wolbachia* within the nematode [7-9].

Filariae are able to maintain a chronic status within the host by influencing regulatory networks and maintaining a strong presence of TGF-β and IgG4 [10-14]. In endemic areas, the majority of *O. volvulus*-infected individuals are classified as generalized onchocerciasis (GEO), these patients present mild pathology, strong regulatory responses including elevated IL-10-producing Tr1 cells and IgG4 but high worm burden and MF loads [12,14-17]. Even within the onchocercomas, the cellular composition consists of Foxp3^+^ regulatory T cells and TGF-β-positive cells [18,19]. GEO patients also present hyporesponsive immune responses when whole blood or isolated PBMCs are stimulated with various antigens - including preparations of *O. volvulus* worm extracts [15,20]. In contrast to infections with *W. bancrofti*, in which only 50% of infected individuals are MF^+^ [21], nearly all *O. volvulus* infections result in the release of MF. The instance of amicrofilaridermic (a-MF) individuals occurs when the worm is still in the prepatent state, the female worms are no longer fecund after many years or the individual has just received or has had repeated rounds of IVM [20,22,23]. In a previous study we demonstrated that MF^+^ and a-MF individuals had different immune responses to filarial-specific or bystander antigens. Moreover, using regression analysis we were able to demonstrate that the responses were influenced by both the individual ivermectin intake (IIT) and the rounds of IVM MDA within the community [20].

Concomitant areas of different filarial and malaria infections vary worldwide and there are currently no precise statistics on the frequencies of onchocerciasis and malaria. Several studies have shown that deworming elicits contrasting effects to bystander responses, including *Plasmodium*. Thus, within this study, we investigated how MF^+^ and a-MF *O. volvulus-*infected individuals responded to MSP-1 (merozoite surface protein-1), which is required by *Plasmodium* to enter the erythrocyte. Moreover, we determined whether these responses were affected by anthelmintic therapy since individuals stemmed from two distinct regions of Ghana that had received differing numbers of IVM rounds. We show that upon co-culture with MSP-1, IL-10, IL-6 and TNF-α secretion by PBMCs from infected individuals were significantly higher than from non-endemic normals (NEN) but this was not the case with regards to IL-5 or IL-13 production. Moreover, MF^+^ individuals produced significantly higher amounts of IL-17A than the a-MF cohort. These findings were then analyzed using a regression analysis employing the covariates MF, IVM therapy and region. The regional covariate denotes the Central and Ashanti regions of Ghana from which individuals resided and at the time of sampling these regions had received 8 and 1 rounds of therapy respectively [24]. We found that the dominant variable in MSP-1 induced responses was the region indicating that bystander responses from *O. volvulus*-infected individuals are influenced by the community intake of IVM.

## Methods

### Study population

210 male and female participants (18–55) were recruited in 2009 as part of the study entitled "Comparison of doxycycline alone vs doxycycline plus rifampicin in their efficacy against onchocerciasis" registered with Current Control Trials as ISRCTN68861628 (http://www.controlled-trials.com/ISRCTN68861628/hoerauf). Individuals resided in 24 villages adjacent to the river Offin in Ghana. These hyperendemic regions for onchocerciasis but not other filarial infections (Ghana MoH-NTD 2007, unpublished findings) were within vector range (<12 km) and included the Upper and Lower Denkyira Districts in the Central Region and the Amansie Central and Adanse South Districts in the Ashanti Region. The data presented here was collated prior to any treatment referred to in the trial protocol. Ethical clearance, including immunological studies, was granted by appropriate ethical committees at the University of Bonn and the University of Science and Technology in Kumasi. Samples from infection-free, non-endemic normal (NEN), volunteers (23–59) were used for comparison. Written informed consent was obtained from all participants.

### Ivermectin therapy and parasitology

Although the Ghanaian Ministry of Health has implemented MDA in the Upper and Lower Denkyira districts (>2001) and in the Amansie and Adanse South areas (>2008) these regions have not been part of either OCP or APOC programmes [24]. Despite the fact that these two regions had had 8 and 1 rounds of therapy respectively, all individuals in our study had had, on average, an individual IVM intake of 1.5. *O. volvulus*-infected individuals were recruited into the study on the presence of at least one nodule but without severe skin lesions. NEN were negative for MF, had no palpable onchocercomas, and had no pathology related to onchocerciasis. All individuals were screened for the presence of dermal microfilariae (MF/mg skin) as previously described [8,20]. In short, two skin biopsies (1–3 mg) from the buttocks were removed with a corneoscleral (Holth) punch and weighed using a electronic balance. During overnight culture at room temperature in 0.9% NaCl solution, MF emerged from the biopsies. Following microscopic examination [8,20] MF load was calculated per mg skin. All *O. volvulus*-infected and NEN individuals were screened for other intestinal helminths (e.g. schistosomes, *Ascaris*). Infections were diagnosed using standard methods (Kato-Katz, and urine analysis) and 14/164 MF^+^ and 4/46 a-MF individuals were co-infected with either hookworm (n = 13), *Schistosoma mansoni* (n = 2) or *S. haematobium* (n = 4). Individuals were treated with albendazole or praziquantel respectively. Details can be viewed in Additional file 1: Table S1.

### PBMC preparation, *in vitro* cell cultures and cytokine measurements

PBMCs were isolated as previously described [20,25]. For culturing, 2 x 10^5^ PBMCs/well were plated onto 96-well plates (U-shaped, Greiner Bio-One, Frickenhausen, Germany) in RPMI 1640 medium (PAA, Linz, Austria), supplemented with 10% FCS, 2 mM L-glutamine, 50 μg/ml penicillin/streptomycin and 50 μg/ml gentamicin (all PAA). Cells were left un-stimulated or stimulated in triplicate with 0.25 μg/ml of merozoite surface protein (MSP-1) prepared as previously described [26] or anti-CD3/anti-CD28 (10 μg/ml and 2.5 μg/ml) from eBioscience (Frankfurt, Germany). Cultures were incubated for 72 hours at 37°C in 5% CO_2_. Supernatants were then collected and screened for IL-5, IL-13, IL-10, IL-17A, IFN-γ, TNF-α and IL-6 cytokines using R&D Duo sets (R&D Systems, Wiesbaden-Nordenstadt, Germany).

### Statistical analysis

Statistical analyses were performed using the software SPSS (IBM SPSS Statistics 20; Armonk, NY), the PRISM 5 programme (GraphPad Software, Inc., La Jolla, USA) and SAS version 9.2 (SAS Institute Inc. Cary, NC, USA). Since most of the variables did not show a normal distribution, the following tests were chosen: to compare three groups a Kruskal-Wallis-test was performed and, if significant, followed by a Mann–Whitney–*U* test for a further comparison of two groups. For comparisons of continuous parameters the Spearman correlation was used. Here, MSP-induced cytokine responses were correlated with the percentage of neutrophils and levels of IL-5 and ECP (eosinophil cationic protein) in plasma in each individual obtained from the study Arndts et al., [20]. Data was further assessed using a generalized linear model. Initial analysis included parameters such as age, nodule sites, co-infections and gender but no relevant associations were detected.

Since the relevant correlations were factors associated with IVM, MF and region we tightened the analysis and evaluated: "times of individual IVM therapy (IIT)", "IVM intake within the last 12 months", "MF-positivity", "microfilarial density" and the regional covariate "Central: Ashanti". At the time of sampling, villages in the Central region had had 8 years of MDA whereas those in the Ashanti region had had only 1. Individuals from 14 villages (n = 82) comprised the "Central" region whereas 128 people came from 10 villages in the "Ashanti" region. For this model continuous variables were rank-transformed. If more than one covariate was below p < 0.1 following univariable analysis, a further multivariable stage was conducted (p < 0.05).

## Results

### Elevated IL-10 but not Th2 responses by *O. volvulus*-infected individuals to MSP-1 antigen

**Figure 1:**
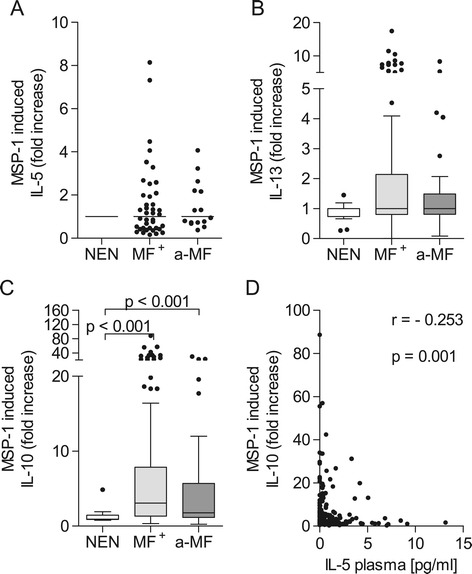
**MSP-1 stimulation alters IL-10 but not Th2 responses.** Isolated PBMCs (2 x 10^5^/well) from NEN (n = 12) or *O. volvulus-*infected MF^+^ (n = 164) or a-MF (n = 46) patients were stimulated with MSP-1 (0.25 μg/ml) for 72 hours. Thereafter, levels of IL-5 **(A)**, IL-13 **(B)** and IL-10 **(C)** were measured in the culture supernatants via ELISA. Data are plotted as fold increase over unstimulated controls. Graphs show box whiskers with median, interquartile ranges and outliers. Statistical significances between the indicated groups were obtained after Kruskal-Wallis and Mann–Whitney tests. **(D)** MSP-1 induced IL-10 levels were correlated with the amount of IL-5 in plasma [20] and tested for statistical significance using the spearman correlation test.

Increased Th2 cytokine production is a hallmark of filarial infections. To test whether these responses were also modulated with regards to *Plasmodium*-derived antigen, freshly isolated PBMCs from MF^+^, a-MF and NEN individuals were co-cultured with MSP-1 for 72 hours. Subsequently, levels of IL-5 and IL-13 were measured in the resulting culture supernatant. No significant differences in these Th2 cytokine responses could be observed between the groups (Figures 1A and B). In contrast, IL-10 was significantly increased in both infected groups when compared to NEN and levels were also principally higher in the MF^+^ group when compared to a-MF individuals (p = 0.08; Figure 1C). Our previous studies demonstrated that IL-5 levels in plasma were significantly elevated in this cohort of MF^+^ individuals [20]. Therefore, we correlated these plasma IL-5 levels with the amount of MSP-1-induced IL-10 and found a significant negative correlation (Figure 1D).

### Compared with NEN, PBMCs from MF^+^ individuals release significantly more TNF-α and IL-6 to MSP-1-antigen

**Figure 2:**
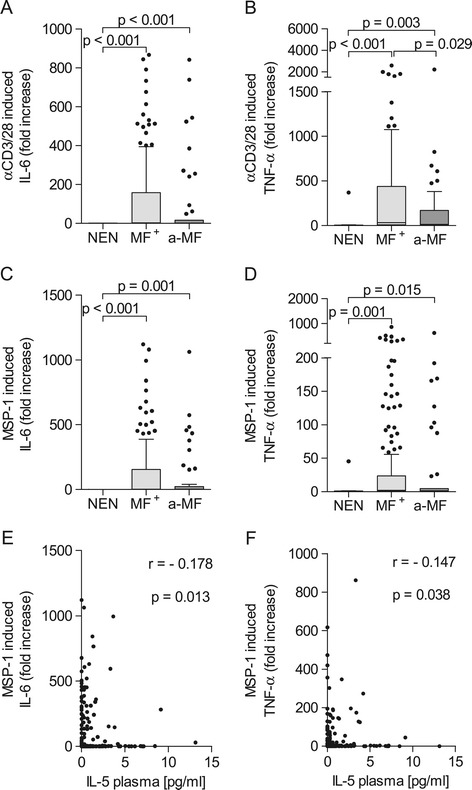
**Elevated innate cytokines in infected individuals.** Isolated PBMCs (2 x 10^5^/well) from NEN (n = 12) or *O. volvulus*-infected MF^+^ (n = 164) or a-MF (n = 46) patients were stimulated with either anti-CD3/anti-CD28 (10 and 2.5 μg/ml respectively, **A** and **B**) or with MSP-1 (0.25 μg/ml, **(C and D)** for 72 hours. Thereafter, levels of IL-6 (A and C) and TNF-α (B and D) were measured in the culture supernatants via ELISA. Data are plotted as fold increase over unstimulated controls. Graphs show box whiskers with median, interquartile ranges and outliers. Statistical significances between the indicated groups were obtained after Kruskal-Wallis and Mann–Whitney tests. MSP-1 induced IL-6 **(E)** and TNF-α **(F)** levels were correlated with the amount of IL-5 in plasma [20] and tested for statistical significance using the spearman correlation test.

IL-6 and TNF-α belong to the innate cytokines, they play important roles in the pathogenesis of malaria [27,28]. Therefore, we investigated their induction after PBMCs from *O. volvulus*-infected and NEN individuals were stimulated with MSP-1. Additionally, PBMCs of all three groups were stimulated with the classical T cell activator anti-CD3/anti-CD28. As depicted in Figure 2, both innate cytokines were significantly up-regulated upon contact with MSP-1 or anti-CD3/anti-CD28 in infected individuals when compared to NEN (Figures 2A to D). In addition, MF^+^ patients produced significantly more TNF-α than a-MF participants after anti-CD3/anti-CD28 stimulation (Figures 2B). However, no significant differences in either IL-6 or TNF-α production to MSP-1 were observed between the infected groups (Figures 2C and D respectively). Nevertheless, MSP-1-induced cytokine levels were negatively correlated (IL-6; r = −0.178 and TNF-α; r = −0.147) with the amount of IL-5 in plasma (Figures 2E and F).

### Up-regulated IL-17A responses to MSP-1 in MF^+^ individuals

**Figure 3:**
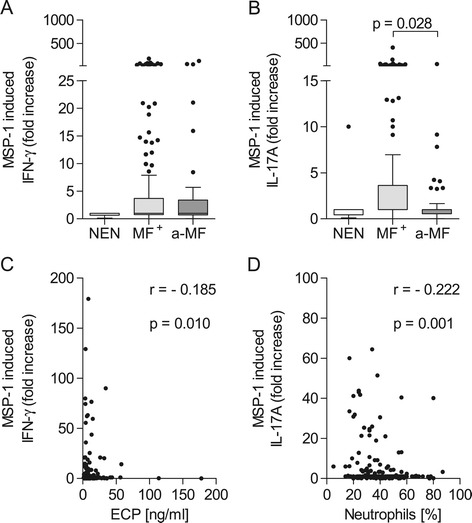
**Up-regulated IL-17A response in MF** ^**+**^ **individuals.** Isolated PBMCs (2 x 10^5^/well) from NEN (n = 12) or *O. volvulus*-infected MF^+^ (n = 164) or a-MF (n = 46) patients were stimulated with MSP-1 (0.25 μg/ml) for 72 hours. Thereafter, levels of IFN-γ **(A)** and IL-17A **(B)** were measured in the culture supernatants via ELISA. Data are plotted as fold increase over unstimulated controls. Graphs show box whiskers with median, interquartile ranges and outliers. Statistical significances between the indicated groups were obtained after Kruskal-Wallis and Mann–Whitney tests. **(C)** MSP-1 induced IFN-γ levels were correlated with the amount of ECP in plasma [20] and **(D)** MSP-1 induced IL-17 responses were correlated with the percentage of neutrophils [20]. Both correlations were tested for statistical significance using spearman correlation test.

Next, we evaluated the release of IFN-γ and IL-17A following MSP-1 stimulation. The production of IFN-γ was not significantly altered between the groups (Figure 3A). In contrast, IL-17A was significantly up-regulated in the MF^+^ group when compared to a-MF patients but not the NEN cohort (Figure 3B). Previous immune profiling of this cohort revealed that MF^+^ individuals had elevated levels of ECP and reduced numbers of peripheral neutrophils [20]. Here we demonstrate that levels of plasma derived ECP negatively correlated (r = −0.185) with the amount of IFN-γ secreted by PBMCs following MSP-1 stimulation (Figure 3C). Furthermore, the percentage of neutrophils was negatively correlated (r = −0.222) to MSP-1 induced IL-17A release (Figure 3D).

### Multivariable regression analysis reveals that immune responses to MSP-1 is mainly associated with ivermectin therapy at the community level

As already described [20,23] the occurrence of a-MF patients is considered to be the result of repeated IVM treatment and/or missing re-infections. When compared to the Ashanti region, at the time of sampling, MDA programmes had run 7 years *longer* in the Central region. To gain insight into whether the previous intake of IVM on an individual level or the frequency of IVM distribution within the community was associated with MSP-1 induced immunological responses, we performed a multivariable regression analysis which included the variables “MF-positive”, “MF/mg”, “times of individual IVM therapy (IIT)”, “IVM in the last 12 months” and “Central: Ashanti” as already described in Arndts et al., [20]. The results of this MSP-1 based regression analysis are shown in Table 1. Levels of IL-5, IL-13 and IFN-γ that were released upon MSP-1 stimulation were not associated with any of the tested covariates. MSP-1 induced IL-10 production was associated with the regional (C:A) and MF^+^ covariates with region being the most associated factor. This implies that IL-10 secretion of PBMCs isolated from individuals in the Central region was lower than those from patients in the Ashanti region. Although IL-17A and TNF-α responses to MSP-1 were associated with all covariates except MF/mg, again the most associated covariate was C:A. In contrast, TNF-α responses to anti-CD3/anti-CD28 were only associated with the region and this was also the case with both IL-6 responses. As shown previously, the frequency of IVM distribution within the community was associated with immune responses and expanding on those findings we demonstrate here that this also influences bystander responses to *Plasmodium-*derived antigens.

**Table 1:** **Regression analysis between MF** ^**+**^ **and a-MF patients**

**Parameter**	**Stimulus**	**Associations with univariable analysis**					**Multivariable analysis**
		**C:A**	**MF/mg**	**MF** ^**+**^	**IIT**	**IVM-12**	
**IL-5**	MSP-1	X	X	X	X	X	---
**IL-13**	MSP-1	X	X	X	X	X	---
**IL-10**	MSP-1	p = 0.0067	X	p = 0.0803	X	X	C:A (C↓A↑, p = 0.0067)
**IL-6**	MSP-1	p = 0.0016	X	X	X	X	nd
	αCD3/αCD28	p = 0.0014	X	X	X	X	nd
**TNF**-α	MSP-1	p = 0.0050	X	p = 0.0224	p = 0.0523	p = 0.0296	C:A (C↓A↑, p = 0.0037)
	αCD3/αCD28	p = 0.0025	X	X	X	X	nd
**IL-17A**	MSP-1	p = 0.0054	X	p = 0.0271	p = 0.0428*	p = 0.0377	C:A (C↓A↑, p = 0.0066)
**IFN-**γ	MSP-1	X	X	X	X	X	---

*denotes negative correlation. 'nd' denotes that only one covariate was below p < 0.1 and therefore no multivariable analysis was done.'---' denotes no associated covariate.

## Discussion

According to epidemiological surveys, there are different frequencies of filarial and malaria co-infections within endemic regions. With regards to *W. bancrofti*, parts of South America, Kenya and Tanzania are reported to have co-infection rates of 3.3%, 4.3%, 11% respectively [29-31]. Surveys of *M. perstans-*infected pregnant women in areas of Uganda were reported to be 18% co-infected with malaria [32]. Nevertheless, a matched prospective study revealed that co-infection with filariae (*W. bancrofti* and *M. perstans*) did not alter susceptibility or severity of acute malaria infection [33]. Currently, a comprehensive overview regarding areas with overlapping *O. volvulus* and *Plasmodium* infections is missing. One study has shown however that in a rural district of Nigeria approximately 8.4% of the community is co-infected [34]. In this study, we demonstrate that *O. volvulus*-infected individuals, residing within co-endemic areas of malaria in Ghana, presented increased IL-6, TNF-α and IL-10 production in response to the *P. falciparum*-derived antigen MSP-1 when compared to uninfected individuals. Th2 responses to MSP-1 on the other hand were comparable in all groups. However, when comparing infected groups, IL-17A responses to MSP-1 were significantly higher in individuals that were MF^+^. Such elevations were unique to MSP-1 since exposure to filarial (*O. volvulus* or *Brugia malayi*) antigens or other bystander antigens (PPD or LPS) were comparable between the groups [20]. Regression analysis further revealed that these MSP-1 induced IL-17A responses were associated with all covariates except microfilarial load but were most strongly associated with the regional covariate. Since this IL-17A response was lower in individuals from the Central region, who had had more rounds of MDA, our data indicate that such lowered infection pressure influences bystander responses to *Plasmodium*-derived antigens. This further indicates that immune responses to *Plasmodium* antigens are modulated even if community members did not regularly participate in therapy.

Interestingly, studies have shown that functional T cell (e.g. IL-17A) responses and the balance between effector and regulatory T cell subsets in the skin relies on signals from commensal microbiota [35] and furthermore changes in gut microbiota have no effect on cutaneous immune homeostasis [36]. Indeed, reconstitution of germ-free mice with *Staphylococcus epidermidis* restored protective immune responses during *Leishmania major* infection. Moreover, this cutaneous commensal exerted its effect through IL-1 signaling which is required for effector T cell function including IL-17A T cells [36]. This raises the hypothesis therefore whether *Wolbachia*, released from dying MF, could modulate IL-17A responses directly in the skin. In association, we recently observed elevated Th17 but decreased Treg cells in individuals with hyper-reactive forms of onchocerciasis, revealing a further association between severe skin pathology and IL-17 effector T cells [37]. With regards to lymphatic filariasis, re-stimulation of PBMCs with filarial antigen revealed elevated expression of IL-17A and F in individuals with severe forms of pathology [38]. When comparing homeostatic levels of IL-17A-producing CD4^+^ T cells, Metenou et al., observed elevated frequencies in individuals infected with filariae (*W. bancrofti* and *M. perstans)* compared to non-infected persons [10]. In another study with the same groups, no differences in IL-17A production could be observed after stimulation with Mal-Ag (*Plasmodium falciparum* schizont extract), *Brugia*-derived antigen or SEB (*Staphylococcus aureus* enterotoxin B) [39]. In association, we previously revealed that PBMC cultures from asymptomatic MF^+^ and MF^**−**^*W. bancrofti*-infected individuals produced very little, if any, IL-17A to *Brugia malayi*-derived antigen or LPS. Significantly elevated levels of IL-17A were released however from cultures of MF^−^ individuals upon activation with anti-CD3/anti-CD28. This was also observed with MSP-1 albeit very few individuals responded [25]. Using flow cytometry it was further observed that the net frequency of IL-17A-producing CD4^+^ T cells in asymptomatic *P. falciparum-*infected individuals increased upon stimulation with Mal-Ag but if individuals were co-infected with filariae (*W. bancrofti* and *M. perstans*) levels did not change [40]. Although no studies have compared *Plasmodium vivax* infections with filarial co-infections it has been shown that the number of IL-17 producing CD4^+^ T cells is significantly increased during uncomplicated acute vivax malaria and directly correlated with elevated IFN-γ producing cells as well [41]. The presence of both IL-17 and IFN-γ in *P. vivax* individuals would create a pro-inflammatory environment and consequently improve responses to the parasite. Indeed, it has been shown in mouse models of malaria that IL-17A may have a protective role since such infected IL-17 deficient mice had higher levels of parasitemia and shortened survival rates [42]. Thus, one could speculate that increased levels of MSP-1 induced IL-17A in MF^+^*O. volvulus*-infected individuals could be beneficial in terms of effective immune responses against malaria re-infection. We further observed a negative correlation between MSP-1 induced IL-17A responses and the percentage of neutrophils in blood. The role of neutrophils during malarial pathogenesis is still under debate but some studies have shown that neutrophil infiltration, activation, and dysfunction may worsen the infection outcome or instigate a predisposition to secondary bacterial infections [43]. Indeed, Chen et al. demonstrated a fundamental role for neutrophils during the pathogenesis of murine ECM by modulating Th1 cytokine expression [44]. Extrapolating those findings to our current study may indicate that such IL-17A responses by MF^+^*O. volvulus-*infected individuals may be protective against malaria infection.

Although most studies have concentrated on *P. falciparum* infections, a study from Winkler and colleagues observed immune differences between MF^+^ and MF^−^ loiasis patients with a small cohort of individuals co-infected with *P. malariae*. They showed that in comparison to amicrofilaremic individuals, MF^+^ patients had strong CD4^+^ Th2 cell responses although the frequency of IL-5 secreting cells and CD8^+^ T cell populations remained equal. The immune profiles of co-infected individuals were on a par with the MF^−^ group [45]. With regards to lymphatic filariasis, no differences in the frequency of IL-5^+^or IL-4^+^ secreting CD4^+^ T cells were observed between *P. falciparum-*infected and co-infected individuals after Mal-Ag stimulation [40] but the authors did observe decreased frequencies of IFN-γ producing cells in the co-infected group [40]. In response to MSP-1, our previous LF study showed no differences in either IFN-γ, IL-5 nor IL-13 between MF^+^ and MF^−^ groups. PBMCs from MF^+^ individuals did however produce reduced amounts of MSP-specific TNF-α, IL-10 and IL-6 [25]. In association, decreased malaria-specific IFN-γ secretion by individuals with patent infections was shown to be IL-10 dependent [39]. Our data here provide evidence that MSP-1-induced IL-10, IL-6 and TNF-α responses are negatively correlated with IL-5 levels in plasma. The role of IL-5 in the context of malaria has so far been studied to a very limited extent and needs further investigation. Nevertheless, a study from Prakash et al. has shown that high levels of plasma IL-5 were found to be associated with a mild form of malaria [46]. In the present study, levels of MSP-1 induced IFN-γ were also negatively correlated to plasma levels of ECP and the latter one was described to be increased in patients with cerebral compared to uncomplicated malaria [47]. Adu et al. claimed in their study a so far unrecognized role for eosinophils in cerebral malaria, which also involves ECP. IL-5 is a potent activator of eosinophils and together with elevated IgE levels is typically associated with cerebral malaria [48]. Currently, the most effective factors in preventing malaria infection are considered to be an early peak of IFN-γ and TNF-α but these cytokines have also been associated with the development of more serious pathology [49,50]. Our findings here demonstrate that proficient IL-17A responses to *Plasmodium* antigens in *O. volvulus*-infected patients are dependent on the presence of MF. Moreover, since an amicrofilaridermic state is a consequence of repeated MDA therapy we further reveal that these IL-17A responses are reduced in communities that have had more IVM therapy but in addition overspill onto individuals that have not received IVM themselves.

## Conclusions

We show here that IL-17A responses to a *Plasmodium*-derived antigen were exclusively elevated in *O. volvulus*-infected individuals that have not received many rounds of MDA (8 rounds in the Central versus 1 round in the Ashanti region). This indicates, therefore, that immune responses to bystander antigens are not only modulated by helminth co-infections *per se* but are also altered by the amount of antihelmintic therapy taken on a community level. Much debate surrounds the issue as to whether deworming in malaria endemic areas is beneficial or not especially when vector biology strongly determines which parasite is the primary or secondary agent. Such findings need to be addressed or taken into consideration at the community level when commencing with MDA programmes.

## Additional file

Additional file 1: Table S1. All participants were screened for other intestinal helminths by standard diagnosis. * denotes one individual that had a dual infection.

## Abbreviations

a-MF: Amicrofilaridermic
GEO: Generalized Onchocerciasis
IIT: Number of times an individual had received IVM therapy
IVM: Ivermectin
MDA: Mass Drug Administration
MF: Microfilariae
MSP-1: Merozoite surface protein-1
NEN: Non-endemic normals
