# Supplementary material for: Reductions in microfilaridermia by repeated ivermectin treatment are associated with lower Plasmodium-specific Th17 immune responses in Onchocerca volvulus-infected individuals
Source: Parasit Vectors. 2015 Mar 28;8:184. doi: 10.1186/s13071-015-0786-5 (PMC4391604; doi:10.1186/s13071-015-0786-5)
Supplement: Additional file 1: Table S1. — All participants were screened for other intestinal helminths by standard diagnosis. * denotes one individual that had a dual infection. [file 13071_2015_786_MOESM1_ESM.docx]

**Table S1**

|  | MF^+^ (n=164) | a-MF (n=46) | NEN (n=12) |
| --- | --- | --- | --- |
| *S. mansoni* | 2 | 0 | 0 |
| *S. haematobium* | 3* | 1 | 0 |
| Hookworm | 10* | 3 | 0 |

All participants were screened for other intestinal helminths by standard diagnosis. * denotes one individual that had a dual infection.
